# State of the Art of Primary PCI: Present and Future

**DOI:** 10.3390/jcm14020653

**Published:** 2025-01-20

**Authors:** Andrea Mignatti, Julio Echarte-Morales, Matteo Sturla, Azeem Latib

**Affiliations:** Montefiore-Einstein Center for Heart and Vascular Care, Montefiore Medical Center, Albert Einstein College of Medicine, 111 East 210th Street, Bronx, New York, NY 10467, USA; juliocecharte@gmail.com (J.E.-M.); msturla@montefiore.org (M.S.); mlatib@montefiore.org (A.L.)

**Keywords:** review, primary PCI, STEMI

## Abstract

Primary percutaneous coronary intervention (PCI) has revolutionized the management of ST-elevation myocardial infarction (STEMI), markedly improving patient outcomes. Despite technological advancements, pharmacological innovations, and refined interventional techniques, STEMI prognosis remains burdened by a persistent incidence of cardiac death and heart failure (HF), with mortality rates plateauing over the last decade. This review examines current practices in primary PCI, focusing on critical factors influencing patient outcomes. Moreover, it explores future developments, emphasizing the role of microvascular dysfunction—a critical but often under-recognized contributor to adverse outcomes, including incident HF and mortality, and has emerged as a key therapeutic frontier. Strategies aimed at preserving microvascular function, mitigating ischemia–reperfusion injury, and reducing infarct size are discussed as potential avenues for improving STEMI management. By addressing these challenges, the field can advance toward more personalized and effective interventions, potentially breaking the current deadlock in mortality rates and improving longer-term prognosis.

## 1. Introduction

The sophisticated contemporary interventions provided to patients experiencing ST-segment elevation myocardial infarction (STEMI) epitomize some of the most remarkable technical advancements in medicine. From the initial landmark trials on thrombolytics, which demonstrated significant reduction in hospital mortality (47% reduction in in-hospital mortality in patients treated within 1 h [1]), to the first angioplasty trials with further reduction in the composite endpoint of in-hospital mortality or reinfarction and significant mortality benefits at 6 months and 2 years compared to thrombolytics [2,3], leading to the introduction of bare metal stents (BMS) and subsequent development of drug eluting stents (DES), the progress of interventional cardiology has impacted the prognosis of STEMI patients to an extraordinary extent.

From the earliest observations of coronary thrombosis to the development of modern drug-eluting stents, technical advancements and clinical trial evidence have transformed the management of acute myocardial infarction (MI). Today, primary percutaneous coronary intervention (PCI) remains the standard of care for patients with STEMI, offering rapid reperfusion and improved clinical outcomes.

The goal of this review is to highlight the current practices adopted when performing primary PCI (pPCI) and to explore possible future developments in this field.

## 2. Epidemiology

Despite the incredible improvement in prognosis, STEMI continues to pose a substantial health challenge in both industrialized and developing nations. In 2018, the United States recorded over one million hospitalizations due to acute coronary syndrome (ACS), with 104,208 mortalities attributed to MI in 2019 [4].

The short-term mortality rate for STEMI patients ranges from 5% to 6% during the initial hospitalization and 7% to 18% at one year [5]. In high income European countries with a fully implemented pPCI strategy, the mortality in the first 30 days decreased substantially between 2003 and 2018 from 10.8% to 7.7% [6].

While the case fatality rate for STEMI has experienced a considerable decline over the past 30 years, it has plateaued in the last decade. A substantial proportion of patients with STEMIs develop heart failure (HF) which triplicates the risk of dying [7]. In a recent analysis by Faridi et al., among 337,274 patients with acute MI (AMI) and no history of HF, 8.0% developed incident HF within 1 year after discharge and 18.8% developed HF within 5 years [8].

The highest risk of ischemic complications following MI occurs within 180 days, after which the risk assumes a relatively linear trajectory [5]. Furthermore, advancements have been observed in terms of hospital stay duration; the mean length of stay has diminished from 3.3 days in 2005 to 2.7 days in 2014, and hospitalizations with a stay exceeding three days declined nearly 50% within the same timeframe [9].

Substantial geographic disparities persist in the availability of perfusion treatment. The utilization of pPCI across Europe varies markedly, ranging from 23 to 884 pPCI procedures per 1,000,000 inhabitants [10]. Geographic discrepancies are also evident in terms of mortality outcomes [10]. Numerous studies have also documented variations in mortality outcomes contingent on patient income status [11].

Furthermore, sex disparities in STEMI outcomes have also underscored deficiencies in STEMI care [12]. Women constitute an equal proportion of STEMI patients aged 75 and older [13]. They frequently exhibit atypical presentations and are more likely to present at an extended time from symptom onset, while concurrently receiving fewer PCI procedures [14,15,16]. Perhaps sex- and gender-related biases contribute to suboptimal outcomes in women; however, robust evidence is lacking due to the underrepresentation of women in large, randomized trials [17,18].

Despite this, remarkable progress has been made in reducing the case fatality rate of STEMI over the past 30 years, there is a clear need for continued efforts to address the disparities in access to care, treatment, and outcomes. By targeting the underlying causes of these variations, such as geographic availability, socioeconomic factors, and sex-specific disparities, the medical community can strive to further enhance the management and prognosis of STEMI patients, ultimately paving the way for equitable and optimal care for all individuals afflicted with this critical health challenge.

From a pathophysiology perspective, one of the unmet needs in STEMI care is to gain an understanding of the mechanisms underlying microvascular obstruction (MVO) and to introduce therapeutic measures aimed at preserving and restoring normal microvascular circulation.

## 3. Prognostic Implications of Door to Balloon Time and Ischemic Time

The concept of “time is muscle”, indicating the correlation between duration of coronary occlusion and myocardial damage/necrosis, has been well demonstrated both in animal studies and in humans. From the first animal model studies demonstrating that coronary occlusion longer than 90 min is associated with death of approximately half of the subtended myocardium [19,20], data from fibrinolysis trials [21], data from sub analysis of angioplasty randomized controlled trials (RCTs) [22] and more recent data [23], the time dependency of revascularization has been very well established.

## 4. STEMI Network System

To provide mechanical reperfusion within the shortest time period to an increasing number of patients, the concept of networking was introduced.

STEMI networks typically consist of a PCI center serving as the hub, non-PCI hospitals functioning as spokes, and emergency medical systems centrally coordinated. Coordinating centers are responsible for creating predefined transportation and treatment protocols, training programs, and quality control. Selection of pPCI centers based on center volume and regional location are important considerations for the network as both have important implications on outcomes [24].

Data from the EORP (EURObservational Research Programme) STEMI Registry has shown that introducing dedicated STEMI networks improves mechanical reperfusion rates in STEMI and influences outcome, with the pPCI rate rising from approximately 20% (EuroHeart Survey ACS Registry 2001) to roughly 80% (EORP STEMI Registry 2015–2018), and in-hospital mortality decreasing from around 7% to about 4% [25]. Currently, the “Stent-Save a Life” program is ongoing, with the primary objective of extending this concept globally according to increasing needs and adapting it to the specific demands of different regions around the world.

## 5. Guideline-Directed Metrics

The ESC/EACTS (European Society of Cardiology/European Association for Cardio-Thoracic Surgery) Guidelines on myocardial revascularization, recommend that the pre-hospital management of STEMI patients be based on regional networks designed to deliver reperfusion therapy expeditiously and effectively, with a focus on offering pPCI as the preferred method of reperfusion to as many patients as possible (recommendation Class I; level of evidence B) [26].

European and American societies strongly recommend reperfusion therapy in all STEMI patients with related symptoms < 12 h [9,26]. Mechanical reperfusion with a pPCI strategy is preferred over fibrinolysis given PCI can be performed within 120 min from diagnosis [26]. When the point from first medical contact to PCI is anticipated to surpass the time window, reperfusion using fibrinolytics is recommended [26]. Primary PCI should be performed within 90 min after first medical contact in transferred patients and within 60 min in patients presenting directly to a PCI center [26].

Studies have demonstrated a clear relationship between time delay to PCI treatment and 1-year mortality in pPCI patients, with each 30 min delay increasing the relative risk by 7.5% at 1 year follow-up [27]. Recent analysis confirmed that adjusted in-hospital mortality was lower for those treated within target times vs. beyond guideline-directed time goals [28].

## 6. Vascular Access

Several studies support the use of the radial approach as the preferred access site for pPCI in ACS patients. The MATRIX (Minimizing Adverse Hemorrhagic Events by Transradial Access Site and Systemic Implementation of angioX) study found that radial access was associated with a significant reduction in all-cause mortality and lower incidence of access site bleeding, vascular complications, and transfusion [29]. Similarly, the RIVAL (Trial of Transradial versus Trans-femoral Percutaneous Coronary Intervention Access Site Approach in Patients with Unstable Angina or Myocardial Infarction Managed with an Invasive Strategy) trial showed lower mortality rates at 30 days with transradial access in a prespecified subgroup of patients with STEMI [30].

The RIFLE-STEACS (Radial Versus Femoral Randomized Investigation in ST-Elevation Acute Coronary Syndrome) study compared transradial and transfemoral approaches in STEMI patients and found that transradial access was associated with lower rates of death, MI, stroke, and major bleeding at 30 days [31]. These findings support the use of a transradial approach as the preferred vascular access for PCI in the STEMI setting.

In recent years, a newer approach via the distal radial artery (anatomical snuffbox access) was proposed. A metanalysis conducted by Ferrante et al. concluded that distal radial artery is associated with lower risks of radial artery occlusion and hematomas, but it requires more time for cannulation and sheath insertion, more puncture attempts, and higher crossover rate [32].

## 7. Percutaneous Coronary Intervention

### 7.1. Stents

In the last two decades, there has been a focused effort to improve stent technology, including design, drug, and polymer, resulting in better outcomes compared to the first generation of DES and BMS, with lower risks of stent thrombosis and recurrent MI. The COMFORTABLE AMI (Comparison of Biolimus Eluted From an Erodible Stent Coating With Bare Metal Stents in Acute ST-Elevation Myocardial Infarction) and EXAMINATION (clinical Evaluation of the Xience-V stent in Acute Myocardial INfArcTION) trials demonstrated that new-generation DES are more effective than BMS in treating AMI due to a lower need for reintervention [33,34]. Long-term follow-up of the EXAMINATION trial have confirmed the superiority of DES over BMS in terms of both patient-related and device-related cardiovascular adverse events [33,35]. In the NORSTENT (Norwegian Coronary Stent) trial (26% STEMI patients), DES were associated with lower rates of definite stent thrombosis and repeat revascularization [36].

Second- and third-generation DES incorporate thinner struts, biocompatible or biodegradable polymers, and improved drug-release kinetics that promote more rapid endothelialization and lower the risk of very late stent thrombosis [37]. Large registry studies and meta-analyses have consistently shown low rates of stent thrombosis, target lesion failure, and other adverse cardiac events with these devices, reinforcing their favorable safety profile in STEMI settings [38]. These technological advancements also allow for more flexible dual antiplatelet therapy regimens, further enhancing the balance between safety and efficacy in select patient populations [39].

### 7.2. Drug-Coated Balloons

Drug-coated balloons (DCB) represent an attractive therapeutic option for STEMI patients as they allow for treating culprit lesions without implanting a permanent vascular scaffold. The randomized REVELATION (REVascularization With PaclitaxEL-Coated Balloon Angioplasty Versus Drug-Eluting Stenting in Acute Myocardial InfarcTION) trial showed that in the setting of STEMI, a DCB strategy was non-inferior to a DES strategy in terms of fractional flow reserve assessed at 9 months [40].

In this study, a total of 120 patients with a non-severely calcified culprit lesion in a native coronary artery and a residual stenosis of <50% after predilation were randomized to treatment with DCB or DES [40]. The primary clinical endpoint was the occurrence of major adverse cardiac events.

Long term follow up at 2 and 5 years confirmed the safety of this technique with no difference in MACE rates between DCB and DES [41].

Larger studies are required to assess the safety and efficacy of this strategy, which appears very promising, particularly for the treatment of complex or calcified lesions where stent deployment in the acute setting is challenging and prone to short- and long-term complications.

### 7.3. Direct Stenting Versus Predilatation

In the context of STEMI, direct stenting without predilatation has advantages over predilatation and stenting, including a reduced risk of complications and improved myocardial perfusion [42]. A post hoc analysis of the HORIZONS-AMI (Harmonizing Outcomes with Revascularization and Stents in Acute Myocardial Infarction) trial demonstrated that direct stenting was successful in the majority of patients and was associated with better ST-segment resolution and lower rates of all-cause death and stroke at the 1-year follow-up [43].

### 7.4. Delayed Versus Immediate Stent Implantation

The delayed stent implantation strategy has been proposed to reduce the risk of no-reflow phenomenon in STEMI patients, but observational trials have shown mixed results [44]. While some studies reported lower no-reflow rates with delayed stent implantation, others, including the DANAMI 3-DEFER (The Third Danish Study of Optimal Acute Treatment of Patients with ST-segment Elevation Myocardial Infarction: Deferred Stent Implantation in Connection with Primary PCI) trial, MIMI (Minimal Invasive Procedure for Myocardial Infarction), and INNOVATION (Impact of Immediate Stent Implantation Versus Deferred Stent Implantation on Infarct Size and Microvascular Perfusion in Patients With ST-Segment-Elevation Myocardial Infarction) trials, did not find any benefits [45,46,47]. A meta-analysis also indicated no improvement in complication rates with delayed stent implantation, leading to the recommendation against its use in pPCI for STEMI patients [48].

A delayed stent implantation may play a role in patients presenting with high thrombus burden, and/or in the setting of a delayed STEMI presentation where the slow flow/no reflow phenomenon might be prevented. While a routine deferral strategy has not been proven to be beneficial, this approach might be beneficial to a particular subset of patients such as those with high thrombus burden, longer lesions, and with high-risk features for slow flow as described by Pradhan et al. [49].

## 8. Thrombectomy

In most STEMI patients, intracoronary thrombus can be detected. Distal embolization has been observed in 5% to 10% of cases, which can worsen outcomes by causing obstruction [50]. To prevent or reduce the risk of distal embolization, various mechanical and manual thrombectomy devices have been proposed.

However, two large RCTs, TOTAL (Trial of Routine Aspiration Thrombectomy with PCI versus PCI Alone in Patients with STEMI) and TASTE (The Thrombus Aspiration in ST-Elevation Myocardial Infarction in Scandinavia), failed to show any differences in clinical outcomes between thrombectomy and PCI alone [51,52]. The TOTAL trial identified a safety issue associated with a higher risk of stroke in patients who underwent thrombectomy compared to those who underwent PCI alone [52]. As a result, thrombus aspiration is not recommended as a routine strategy in STEMI patients treated with pPCI, but it may be considered in patients with high thrombotic load after vessel recanalization [26].

The use of mechanical devices might prove beneficial in a selected group of patients and initial studies are encouraging this, as demonstrated in the CHEETAH (A Prospective, Multicenter Study to Evaluate the Safety and Performance of the CAT RX Aspiration Catheter in Patients With a High Thrombus Burden Acute Coronary Vessel Occlusion) study [53], where sustained mechanical aspiration before PCI in high thrombus burden cases was safe and associated with high rates of thrombus removal. An RCT is being developed to further elucidate the effects of this therapy.

## 9. Pharmacotherapy

During primary angioplasty for AMI, antiplatelet and anticoagulant therapies play a critical role in preventing thrombotic complications. Antiplatelet therapy is aimed at inhibiting the formation of platelet aggregates and promoting platelet disaggregation, while anticoagulant therapy is intended to prevent the formation of blood clots by inhibiting the coagulation cascade.

### 9.1. Antiplatelet Therapy

The most commonly used antiplatelet agents during primary angioplasty are aspirin and P2Y12 receptor inhibitors, such as clopidogrel, prasugrel, and ticagrelor. Prasugrel or ticagrelor are the preferred P2Y12 inhibitors due to their faster onset, higher potency, and better clinical outcomes compared to clopidogrel [54,55]. Cangrelor is an intravenous P2Y12 inhibitor that rapidly and effectively reduces platelet aggregation. It has been shown to be effective in reducing the risk of stent thrombosis and major adverse cardiovascular events in patients undergoing PCI. The CHAMPION (Cangrelor versus Standard Therapy to Achieve Optimal Management of Platelet Inhibition) trials, which compared cangrelor to other antiplatelet agents, demonstrated its efficacy in reducing the rate of ischemic events without increasing bleeding complications [56,57,58]. The findings of the PITRI (Platelet Inhibition to Target Reperfusion Injury) trial were recently released [59]. This phase 2 trial was multicenter, randomized, double-blind, and placebo-controlled, taking place between November 2017 and November 2021 across six cardiac centers in Singapore [59]. The study assessed the effects of administering cangrelor during pPCI on patients with STEMI, who were also receiving oral ticagrelor, on MI size and MVO [59]. The results indicated that cangrelor did not significantly reduce the size of MI or prevent MVO, despite achieving a notable decrease in platelet reactivity during the PCI procedure [59].

### 9.2. Intravenous Glycoprotein IIb/IIIa Inhibitors

The use of glycoprotein IIb/IIIa receptor inhibitors in patients with ACS has shown limited benefit in improving clinical outcomes and may increase bleeding complications. The use of these inhibitors is generally reserved for patients with a large thrombus burden or no-reflow.

Both American and European guidelines do not routinely recommend the use of glycoprotein IIB/IIIA inhibitors; however, they advise their use in patients with large thrombus burden, no-reflow, or slow flow (Class of recommendation IIa; level of evidence C) [9,26]. The REVERSE-FLOW (Glycoprotein IIb/IIIa Inhibitors VERsus Standard Therapy in PatientS with Myocardial Infarction and Angiographic Evidence of No-reFLOW) trial failed to prove that bailout GP IIb/IIIa inhibition in AMI patients with angiographic MVO reduced infarct size [60]. However, a reduction in cardiac magnetic resonance (CMR)-derived MVO was observed, which was associated with an increase in non-fatal bleeding events [60].

### 9.3. Anticoagulants

In addition to antiplatelet therapy, anticoagulant therapy is also necessary during primary angioplasty. Unfractionated heparin (UFH) is the most commonly used anticoagulant agent, as it has a rapid onset of action and can be easily monitored using activated clotting time. Nevertheless, UFH has several limitations, including a narrow therapeutic window and the potential for heparin-induced thrombocytopenia. Low-molecular-weight heparins have also been used in primary angioplasty and have been shown to be as effective as UFH with a lower risk of bleeding.

Bivalirudin use was studied extensively and a metanalysis of dedicated randomized trials comparing bivalirudin with UFH showed similar mortality with bivalirudin with a decrease in major bleeding rate and an increased risk of acute stent thrombosis [61]. The MATRIX (Minimizing Adverse Hemorrhagic Events by Transradial Access Site and Systemic Implementation of Angiox) trial included more than 7000 patients with ACS (56% with STEMI) and compared bivalirudin with UFH [61]. The primary endpoint (composite of death, MI, or stroke) was similar in both groups; however, patients in the bivalirudin group showed lower total and cardiovascular mortality, bleeding, and an increase in definite stent thrombosis [61]. A recent study on the use of Bivalirudin plus a high dose infusion post PCI compared with heparin reported that in patients with STEMI undergoing pPCI predominantly with radial access and bivalirudin, plus a high dose infusion, significantly reduced the 30-day composite rate of all-cause mortality or major bleeding [62].

In the setting of conflicting results, more studies, including cost effectiveness analysis, are required to assess the clinical benefits of bivalirudin in pPCI compared to heparin.

## 10. Special STEMI Situations

### 10.1. Cardiogenic Shock and Hemodynamic Support Devices

Patients with STEMI complicated by cardiogenic shock are at exceedingly high risk for mortality, making prompt intervention critical. Various percutaneous mechanical circulatory support devices are available to stabilize hemodynamics and potentially improve outcomes (Table 1).

#### 10.1.1. Intra-Aortic Balloon Pump

In non-shock STEMI patients, the routine use of an intra-aortic balloon pump (IABP) did not show either a 30-day survival benefit or improved left ventricular ejection fraction (LVEF) in a metanalysis of randomized trials, while it was associated with higher bleeding and stroke rates [63]. The CRISP AMI (Counterpulsation to Reduce Infarct Size Pre-PCI Acute Myocardial Infarction) study also did not support the routine use of IABP in patients with anterior STEMI without shock [64].

In the IABP-SHOCK II (Randomized Clinical Study of Intraaortic Balloon Pump Use in Cardiogenic Shock Complicating Acute Myocardial Infarction) trial, mortality evaluated at 30 days and 12 month was similar in patients with cardiogenic shock (CS) treated with and without IABP [65]. Currently, based on the available evidence, the ESC clinical practice guidelines contraindicate the routine use of IABP in patients with CS; however, it could be considered in patients with hemodynamic instability/CS due to mechanical complications of MI [26].

#### 10.1.2. Impella

The ISAR-SHOCK (Left Ventricular Assist Device (Impella LP 2.5) vs. Intraaortic Balloon Counterpulsation (IABP) in Patients With Cardiogenic Shock and Acute Coronary Syndromes) trial compared the Impella 2.5 system with IABP in patients with STEMI and CS [66]. The trial showed a significant improvement in the cardiac index in the Impella group compared to the IABP group [66]. However, the secondary endpoints, such as lactic acidosis, hemolysis, and mortality at the 30-day follow-up, did not differ between the two arms [66]. The overall mortality rate in the cohort was high [66].

The DanGer Shock (Danish–German Cardiogenic Shock) trial was the first RCT to show a survival benefit with the use of a microaxial flow pump (Impella device) (45.8% mortality at 180 days in patients randomized to Impella support versus mortality of 58.5% in the standard of care group) [67]. The exclusion criteria were cardiac arrest, right ventricular failure, and out of hospital arrest with poor neurological status on arrival in the ED [67]. The number needed to treat to avoid one death was eight [67]. The benefit of Impella was greater in patients with lower mean blood pressure and multivessel coronary artery disease (CAD) [67]. Patients randomized to the Impella arm experienced higher rates of adverse events (severe bleeding and limb ischemia) [67]. Renal replacement therapy was also higher in the microaxial flow pump; a finding which will need further evaluation [67]. The use of the Impella device for left ventricular unloading will be discussed in the section below.

#### 10.1.3. TandemHeart

The TandemHeart is an extracorporeal ventricular assist device designed to lower left ventricular preload and reduce left atrial volume by diverting blood from the left atrium. This process decreases left ventricular stress and workload while enhancing systemic mean arterial pressure and myocardial perfusion. Thiele et al. documented its use in 18 patients with STEMI and CS, and Kar et al. presented a series involving 117 CS patients where the TandemHeart device quickly mitigated severe hemodynamic instability [68,69]. Despite these reports, experience with this device is limited, and there are no RCTs or extensive registries available.

#### 10.1.4. Extracorporeal Membrane Oxygenation

Although extracorporeal membrane oxygenation (ECMO) is widely utilized in experienced centers, the supporting data for its use in patients suffering from AMI complicated by CS largely consists of small, single-center case series. In a retrospective observational registry at a single center, Sheu et al. compared the clinical outcomes of STEMI patients undergoing pPCI, finding that ECMO-assisted PCI led to improved outcomes at 30-day follow-up [70].

The ECLS-SHOCK trial recently demonstrated that the 30-day death rate was not lower among the patients who received ECLS therapy compared to those who received medical therapy alone in patients with AMI complicated by CS [71].

### 10.2. Multivessel CAD

Non-infarct related CAD is common in STEMI patients. In a retrospective study of 68,000 patients from eight large STEMI trials from 1993 to 2007, 52.8% had obstructive non-infarct related artery (IRA) disease [72]. The unadjusted 30-day mortality rate in patients with non-IRA disease was 4.3%, while in patients without non-IRA disease it was 1.7% [72]. It has also been shown that ST segment resolution is less likely to resolve in patients with a higher burden of disease. The reduced reperfusion success is related to cumulative incidence of death at 1 year according to the presence of single-, double-, or triple-vessel disease (3.2%, 4.4%, and 7.8%, respectively) and revascularization of the non-IRA within 30 days is associated with lower 1 year mortality [73].

The CULPRIT (Complete Versus Lesion-Only Primary PCI Trial) trial concluded that in-hospital complete revascularization of angiographically significant non-IRA lesions resulted in improved clinical outcomes at 12 months compared with treatment of the culprit lesion only [74].

In 2019, the COMPLETE (Complete versus Culprit-Only Revascularization Strategies to Treat Multivessel Disease after Early PCI for STEMI) randomized 4041 patients to PCI of the culprit lesion only vs. multivessel PCI within 45 days [75]. The primary endpoint (cardiovascular death and MI) at 3 years did not show differences between both groups [75]. On the other hand, the secondary endpoint (cardiovascular death, MI and ischemic driven revascularization) was more frequent in patients who underwent PCI of only the culprit lesion [75].

Recently, the MULTISTARS AMI (Multivessel Immediate versus Staged Revascularization in Acute Myocardial Infarction) study demonstrated that among patients in a hemodynamically stable condition with STEMI and multivessel CAD, immediate multivessel PCI was noninferior to staged multivessel PCI with respect to the risk of death from any cause, nonfatal MI, stroke, unplanned ischemia-driven revascularization, or hospitalization for HF at 1 year [76].

It is known that multivessel CAD is common in ACS patients presenting in CS and associated with worse outcomes [77,78]. The CULPRIT-SHOCK (The Culprit Lesion Only PCI versus Multivessel PCI in Cardiogenic Shock) trial evidenced that patients presenting with CS undergoing PCI should undergo infarct-related artery PCI only [79]. When compared to patients that underwent immediate multivessel PCI, culprit only PCI was shown to have a significantly reduced risk of death (0.84 (95% CI, 0.72 to 0.98; *p* = 0.03)) and need of renal replacement therapy (0.71 (95% CI, 0.49 to 1.03; *p* = 0.07)) at 30 days [79].

### 10.3. Late-Presenters

Despite improved STEMI networks and education, late presentation—beyond 12 h from symptom onset—still occurs in 10–15% of STEMI patients [80,81]. Early fibrinolytic trials established the 12 h limit, as mortality benefits were primarily within this window [1,82,83]. However, more recent data challenge this strict cutoff.

Nonetheless, emerging evidence supports extending the window for mechanical reperfusion. The BRAVE-2 (Beyond 12 h Reperfusion Alternative Evaluation-2) trial, though relatively small, demonstrated that PCI performed 12–48 h after symptom onset reduced infarct size and was associated with improved long-term outcomes, including lower mortality at 4 years [84,85]. These findings have influenced guideline recommendations. European guidelines, aligning with BRAVE-2 results and corroborated by large observational analyses, suggest offering PCI up to 48 h after symptom onset in stable patients (Class IIa) [26]. In a recent large, nationwide analysis, latecomers (12–48 h) who underwent revascularization had significantly lower all-cause mortality at both 30 days and long-term follow-up (median 58 months) compared to those who did not undergo PCI [86].

In contrast, American guidelines propose a Class IIa recommendation for PCI in stable STEMI patients presenting 12–24 h after symptom onset, but recommend against routine PCI beyond 24 h (Class III) in stable patients [9]. Both American and European guidelines, however, agree that emergent PCI is always indicated if patients present late with complicating features such as CS, acute HF, or life-threatening arrhythmias [9,26]. Stable patients presenting beyond 24 h (per American guidelines) or beyond 48 h (per European guidelines) are generally managed similarly to those with chronic coronary syndromes [9,26]. The OAT (Occluded Artery Trial) and DECOPI (Desobstruction Coronaire en Post-Infarctus) trials support this approach, having demonstrated no benefit from routine very-late PCI in stable patients [87,88].

In the absence of ongoing ischemia or hemodynamic instability, the role of noninvasive imaging to assess myocardial viability may become increasingly important. Identification of viable myocardium can guide the decision to proceed with PCI in stable, late-presenting STEMI patients—particularly those beyond 48 h post-infarct—by determining whether a revascularization strategy has the potential to improve left ventricular function and long-term prognosis. Future well-powered, prospective trials are needed to clarify and refine the potential for viability-guided strategies in the late-presenter population.

### 10.4. Calcific Lesions

Sugiyama et al. conducted a detailed analysis of calcified culprit plaques in patients with ACS [89]. From 1241 patients presenting with ACS who had undergone pre-intervention OCT, 157 (12.7%) patients were found to have a calcified plaque at the culprit lesion [89]. Three distinct types of calcified culprit plaques were identified: eruptive calcified nodules, superficial calcific sheet, and calcified protrusion (25.5%, 67.4% and 7.1%, respectively) [89]. Superficial calcific sheet, which is frequently located in the left anterior descending coronary artery, is the most prevalent type and is also associated with greatest post-intervention myocardial damage [89]. Intravascular coronary lithotripsy is becoming an attractive and promising modality for treatment of severely calcific lesions and could become a valid alternative to rotational, orbital and laser atherectomy particularly in patients presenting with ACSs.

A recent prospective multicenter, real world registry of coronary lithotripsy in calcified coronary arteries analyzed the performance of coronary IVL in calcified coronary lesions in real life, all comers setting [90]. A total of 426 patients were included and 63% of them presented with ACSs [90]. The authors concluded that coronary lithotripsy is a safe procedure in “real life” setting facilitating stent implantation in severely calcified lesions [90].

More studies are necessary in order to elucidate the best timing and strategies for revascularizing calcific lesions in STEMI patients.

## 11. Microvascular Circulation and Obstruction

It is well recognized that microvascular circulation plays a significant role in the pathogenesis of STEMI. MVO is defined as the inability to re-perfuse the coronary microcirculation in a previously ischemic region despite opening of the epicardial vessel and multiple mechanisms are at the basis of its pathogenesis.

While it is well known that infarct size is an independent predictor of adverse left ventricular (LV) remodeling after MI [91], recent studies support the evidence that the extent of MVO also represents a fundamental predictor of LV remodeling and may be more predictive of major adverse cardiovascular events than infarct size itself [92].

Patients presenting with STEMI may develop MVO, with a variable prevalence ranging from 5% up to 60%, according to the methods used to assess the phenomenon and to the population under study [93]. It has also been postulated that due to its dynamic nature, MVO is irreversible in 50% of patients [93].

As described by Niccoli et al. [93] four interacting mechanisms responsible for microvascular dysfunction have been identified: ischemia related injury, reperfusion-related injury, distal embolization, and individual susceptibility of the microcirculation to injury (both genetic and due to pre-existing coronary microvascular dysfunction). Understanding the pathogenesis of each of these mechanisms is fundamental in order to implement new therapeutic approaches.

Ischemia related injury represents a well-known mechanism responsible for cardiomyocyte death, and when ischemia lasts more than 3 h, the adverse effects of ischemia-associated injury are significantly worsened [94]. Furthermore, the formation of interstitial myocardial edema secondary to ischemic injury results in the compression of capillaries and small arterioles, worsening flow through these dysfunctional vessels.

In cardiomyocytes, reperfusion stimulates the production of radical oxygen species which are implicated in calcium overload, mitochondrial swelling, and cell disruption. It has been postulated that ischemia followed by reperfusion may also favor intramyocardial hemorrhage and that the activation of inflammation and coagulation in the setting of STEMI leads to thrombosis, endothelial activation, and consumption of coagulation factors [93].

Distal embolization is also a well-known phenomenon in the pathogenesis of STEMIs [95], and it has been suggested that small number of emboli during pPCI in the setting of STEMI, although not affecting baseline myocardial perfusion, may create a local milieu with release of inflammatory and vasoactive substances from coronary plaque, which have the potential to increase the severity of the functional impairment of the coronary circulation [96,97].

Individual susceptibility to microvascular dysfunction has been described as a possible mechanism causing MVO [98]. Preexisting microvascular dysfunction, particularly in patients with multiple cardiovascular risk factors (diabetes, hyperlipidemia, hypertension) may be associated with an increased risk of developing MVO [98]. The presence of ischemic preconditioning, which not only protects the myocardium but might also protect the coronary microcirculation, is another factor modulating individual susceptibility to MVO [93,98].

### Diagnosis of Microvascular Dysfunction

Diagnostic tools for identifying microvascular dysfunction are classified as invasive (Doppler wire, Thrombolysis in Myocardial Infarction (TIMI) myocardial perfusion grade count, myocardial blush grade), and non-invasive (CMR, positron emission tomography and myocardial contrast echocardiography).

The gold standard method for assessing microvascular function is the direct measurement of coronary blood flow velocity using an intracoronary Doppler wire.

The index of microcirculatory resistance (IMR) is an invasive method that employs dedicated wires to evaluate coronary microvascular function by measuring both coronary pressure and flow [99]. One significant benefit of IMR is its ability to be swiftly and easily measured in the catheterization lab alongside fractional flow reserve (FFR). This simultaneous measurement facilitates independent assessments of the epicardial arteries (via FFR) and the microvasculature (through IMR), enhancing diagnostic precision [100,101].

CMR on the other hand, allows quantification and localization of MVO with high spatial resolution and has been shown to correlate with invasive assessment [100,101,102,103]. De Maria et al. investigated the relationship between the IMR and MVO in patients following STEMI. The study found that while IMR and MVO are correlated, using an IMR threshold of 40 leads to discrepancies between the two measurements in about one-third of cases [100]. Patients with MVO and elevated IMR were observed to have a larger infarct size compared to those with MVO and an IMR ≤ 40 [100]. Additionally, patients with MVO and an IMR ≤ 40 showed significant regression of IS at 6 months, whereas no substantial change in IS extent was observed in patients with MVO and higher IMR [100].

The Controlled Flow Infusion (CoFI™ System) is a new technology with the potential of providing a beat-by-beat evaluation of coronary microvasculature, enabling the calculation of dynamic microvascular resistance by measuring variation in distal coronary artery pressure in response to a predefined infusion rate of crystalloid solution. The MOCA I (Microvascular Obstruction With CoFI™ System Assessment) study, showed that the CoFi system is able to detect MVO at the end of pPCI with good sensitivity and specificity in a small number of patients. Additional validation studies are required to confirm the safety and efficacy of this system [104].

## 12. STEMI Prognosis

A recent meta-analysis involving 2632 patients from 10 RCTs demonstrated a strong association between infarct size—measured by CMR imaging or single-photon emission computed tomography within one month post-pPCI—and the rates of 1-year hospitalization for HF and all-cause mortality [91]. Specifically, each 5% increase in myocardial infarct size corresponded to a 20% rise in the relative hazard ratio for these outcomes within one year [91].

Additionally, for every 1% absolute increase in microvascular dysfunction, there was an independent 14% relative increase in 1-year all-cause mortality and an 8% increase in hospitalization for HF during the same period [91].

Intramyocardial hemorrhage (IMH) is a severe form of MVO that typically develops in the core of the infarct following MVO [105]. It tends to expand over several hours after PCI and is caused by vascular endothelial damage and the accumulation of red blood cells in the myocardial extracellular space. There is ongoing debate about whether IMH is a cause or a consequence of severe ischemic–reperfusion injury. Additionally, some studies have reported that residual iron is frequently observed in patients following STEMI and is associated with adverse left ventricular remodeling [106].

Interestingly, some authors have reported distinct time courses for IMH and MVO following STEMI. IMH has been found to be more closely associated with adverse outcomes compared to MVO. In a cohort of 300 STEMI patients, Carrick et al. observed that IMH typically occurs within the first two days after the index event, whereas MVO peaks between 4 and 12 h post-MI and subsequently decreases [107].

Furthermore, IMH has been linked to more severe MI, systemic inflammation, and adverse LV remodeling. This strong association between IMH and worse LV function during follow-up was also demonstrated by Bulluck et al. in a study of 48 STEMI patients undergoing CMR [106]. Their findings highlight the intriguing concept that IMH and residual myocardial iron could serve as potential therapeutic targets to prevent adverse LV remodeling in reperfused STEMI patients. Additionally, persistent residual iron, alongside IMH, may contribute to the worsening of LVEF, ultimately leading to higher rates of all-cause mortality or HF [108].

## 13. Therapeutic Approaches

Over the years, several pharmacological strategies have been attempted to enhance microcirculatory function, avert reperfusion injury, and diminish infarct size in STEMI patients. These strategies have included intracoronary administration of agents like adenosine, nitroprusside, and abciximab. Despite these efforts, none of these treatments have been proven to improve clinical outcomes [21].

Ischemic preconditioning has demonstrated a protective role in animal studies but had not shown any clinical outcome benefit among STEMI patients undergoing pPCI [21].

## 14. Future Targets for Novels Device-Based Therapies

As described by De Maria et al. [109] in an attempt to improve the outcome of STEMI patients undergoing pPCI, strategies to target the different pathways responsible for microvascular injury are being developed.

Prevention of distal embolization, mitigation of ischemia–reperfusion injury and preservation of microvascular integrity are among the mechanisms that are currently being explored as potential targets for improving outcomes in STEMI patients.

### 14.1. Prevention of Distal Embolization

#### 14.1.1. Thrombus Aspiration

While earlier large-scale randomized trials did not demonstrate a clinical advantage for manual thrombus aspiration, mechanical aspiration devices may hold potential benefits for select patient populations. The suboptimal efficacy of conventional manual thrombectomy in removing thrombi can be attributed to factors such as a disparity between the vessel lumen size and the extraction area, a gradual reduction in aspiration force during the procedure, and the phenomenon known as “wire-biasing” [109].

The CHEETAH study—a prospective multicenter on 400 patients—demonstrated that sustained mechanical aspiration thrombectomy in high thrombus burden ACS patients prior to PCI was safe and was associated with high rates of thrombus removal, flow restoration, and normal myocardial perfusion on final angiography [53]. Additional large-scale, randomized studies will be essential to further validate the promising safety and performance outcomes. These studies will help ensure the reliability and generalizability of the findings, providing a more robust understanding of the intervention’s effectiveness across diverse populations and clinical settings.

#### 14.1.2. Sonothrombolysis

Sonothrombolysis is a novel strategy consisting of creating microbubble-induced cavitations of the thrombus through high mechanical index (HMI) impulses delivered by a conventional diagnostic ultrasound transducer after intravenous infusion of standard ultrasound enhancing agents (e.g., contrast agent).

This concept was tested in 100 STEMI patients randomized 1:1 to sonothrombolysis before and after pPCI or standard pPCI [110]. Angiographic recanalization before pPCI, ST-segment resolution, infarct size by magnetic resonance imaging, and systolic function at 6 months were compared [110]. The results showed that sonothrombolysis added to pPCI improves recanalization rates and reduces infarct size, resulting in sustained improvements in systolic function after STEMI [110]. Sonothrombolysis was associated with reduced infarct size and higher LVEF both after revascularization and at 6-months follow-up [110].

Further research is needed to determine whether a simple non-invasive therapy could be beneficial in large, randomized trials.

### 14.2. Mitigating Ischemia–Reperfusion Injury

#### Mechanical Unloading

Mechanical ventricular unloading refers to any intervention aimed at reducing myocardial oxygen consumption. Multiple preclinical models over the past 20 years have shown that, in comparison with reperfusion alone, mechanically unloading the left ventricle before, not after, coronary reperfusion reduces ischemia–reperfusion injury and myocardial infarct size in AMI [111,112,113].

An initial study using an animal model with a percutaneous left atrial-to-femoral artery bypass pump demonstrated that implementing LV mechanical unloading for 30 min prior to reperfusion resulted in a significant reduction in myocardial infarct size by 40–50% compared to reperfusion alone [114].

Kapur et al. demonstrated that mechanically conditioning the myocardium using an axial flow catheter while delaying coronary reperfusion decreases LV wall stress, increases cardioprotective signaling, reduces apoptosis, and limits myocardial damage in AMI [115].

The DTU-STEMI (Door-To-Unload in STEMI) pilot trial was a prospective, multicenter, randomized pilot trial involving 14 centers in the United States to explore the feasibility, safety, and potential benefit of mechanical unloading before coronary reperfusion in patients presenting with anterior STEMI [113].

Fifty patients with anterior STEMI were randomized to LV unloading by using the Impella CP followed by immediate reperfusion versus delayed reperfusion after 30 min of unloading [113]. The study demonstrated that LV unloading using the Impella CP device with a 30 min delay before reperfusion is feasible within a relatively short time period in anterior STEMI [113].

The results lead to the design of the STEMI-DTU (Primary Unloading and Delayed Reperfusion in ST-Elevation Myocardial Infarction) Pivotal trial, which will compare reperfusion alone versus LV unloading and delayed reperfusion in patients with anterior STEMI (NCT03947619).

### 14.3. Enhancing Microvascular Function/Integrity

#### Supersaturated Oxygen

Supersaturated oxygen (SSO2) therapy refers to the delivery of hyperoxemic blood to the ischemic myocardium after pPCI. It has been shown to reduce endothelial cell edema, induce capillary vasodilatation, and limit infarct size in experimental animal models [116,117].

In the AMIHOT I (Acute Myocardial Infarction with HyperOxemic Reperfusion) trial, O’Neil et al. conducted a randomized study involving 269 patients experiencing anterior or large inferior STEMI with TIMI flow grades 0–2, all undergoing pPCI or rescue PCI [118]. Participants were assigned to receive either 90 min of intracoronary SSO_2_ therapy or standard care [118]. The findings indicated that, among patients with anterior MIs reperfused within six hours, those treated with SSO_2_ therapy exhibited a significant reduction in infarct size at 14 days and showed improved regional wall motion at the three-month follow-up [118].

In the AMIHOT II (Acute Myocardial Infarction With HyperOxemic Therapy II) trial, Stone et al. demonstrated that among patients with anterior STEMI undergoing PCI within 6 h of symptom onset, infusion of SSO2 into the left anterior descending artery infarct territory results in a significant reduction in infarct size with noninferior rates of major adverse cardiovascular events at 30 days [119].

The results of the AMIOT II trial in addition to the IC-HOT (IntraCoronary Hyperoxemic Supersaturated Oxygen Therapy) study in which 100 anterior STEMI patients received hypoxemic blood for 60 min through a diagnostic catheter in the left main coronary artery, lead the US FDA to approve supersaturated oxygen for treatment of patients with anterior STEMI involving the left anterior descending artery undergoing primary pPCI within 6 h of symptom onset [117].

The AMIHOT III (Acute Myocardial Infarction with Hyperoxemic Therapy III) trial (NCT04743245) is an ongoing multi-center randomized study that evaluates the use of intracoronary hyperoxemic supersaturated oxygen therapy for 60 min in anterior AMI patients with successful reperfusion (via PCI) within 6 h of symptom onset. This therapy is compared to standard care. The primary composite endpoint includes the following events: all-cause death, reinfarction, ischemia-driven target vessel revascularization, major or minor TIMI bleeding, new onset HF or rehospitalization for HF, and stent thrombosis (ARC definite or probable).

## 15. Novel Pharmacological Strategies

Currently there are no effective therapies to limit reperfusion injury. FDY5301 is a proprietary, patented therapeutic designed to down-regulate pathologic inflammatory responses following acute and chronic injury. It is composed of sodium iodide, which catalytically destroys hydrogen peroxide and is postulated to limit the detrimental effects of excessive reactive oxygen species during cardiac reperfusion.

A phase 2 study showed that Intravenous FDY-5301, delivered immediately prior to pPCI in acute STEMI, is feasible, safe, and shows potential efficacy with a trend towards reduced final infarct size [120]. Currently, the IOCYTE-AMI 3 trial (NCT04837001) has completed the recruitment phase, and the results are pending release. Briefly, the objective is to assess the effect of FDY-5301 on cardiovascular mortality and acute HF events in subjects with an anterior STEMI undergoing pPCI.

## 16. Artificial Intelligence

The emergence of digital health and artificial intelligence (AI) has the potential to revolutionize clinical care, although real-world patient evaluation has not yet seen transformative changes. Traditional practices like history taking and physical examination remain dominant, but a growing range of AI-enhanced digital tools may soon augment these methods, making the clinical process more data-driven. In a prospective, cluster-randomized clinical trial, AI-ECG-based screening resulted in a 32% higher rate of diagnosing left ventricular systolic dysfunction without significantly increasing echocardiography referrals compared to standard care [121]. Additionally, AI-ECG has shown potential in screening for occult atrial fibrillation [122], various cardiomyopathies [123], electrolyte abnormalities, acute conditions like MI [124,125], and providing insights into heart function, including left and right ventricular performance.

## 17. Conclusions

While pPCI has contributed significantly to the improvement in outcomes of STEMI patients, It is now very evident that STEMI therapy should not only focus on the reperfusion of epicardial vessels but should also address microvascular reperfusion.

MVO pathogenesis is very complex and a multitargeted approach using a combination of therapies may be the most appropriate and effective solution to improve outcomes.

Further studies are required to evaluate if an invasive assessment of microvascular resistance during pPCI may be useful to select patients.

Future research efforts should also be directed to evaluate potential benefits in high-risk subgroups, which are prone to develop MVO in different time windows in the course of MI before, during, and after pPCI.

Any new strategy aimed at improving STEMI outcomes should ideally be incorporated in the current pPCI workflow, without causing excessive delays in reperfusion, and should be as practical as possible in order to be routinely adopted.

## Figures and Tables

**Table 1:** Comparison of Mechanical Circulatory Support Devices used During Ischemic Cardiogenic Shock.

	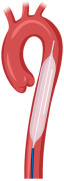 *IABP*	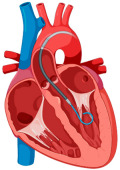 *Impella CP*	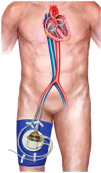 *Tandem Heart*	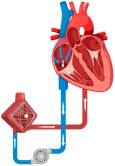 *VA-ECMO*
*Mechanism*	Pulsatile	Axial (Continuous)	Centrifugal (Continuous)	Centrifugal (Continuous)
*Flow* (L/min)	↑ CO 0.5–1.0	Up to 4.3	2.5–5	3–7
*Access* (Fr)	Femoral artery (7–9 Fr)	Femoral artery (14 Fr)	Femoral artery (15–19 Fr) Femoral vein (21 Fr)	Femoral artery (15–19 Fr) Femoral vein (18–24 Fr)
*Support Provided*	Minimal LV support Diastolic augmentation	Partial LV support	Partial to complete LV support	Complete biventricular support
*Anticoagulation*	Not required if augmenting 1:1	Yes	Yes	Yes
*Contraindications*	Significant AR Aortic dissection Aortic aneurysm Significant Iliofemoral disease	Significant AS (AVA ≤ 0.6 cm^2^) Significant AR Mechanical AV LV thrombus Significant Iliofemoral disease	Significant AR LA thrombus Inability to tolerate AC Significant Iliofemoral disease	Significant AR Significant Iliofemoral disease

AR: aortic regurgitation; AS: aortic stenosis; CO: cardiac output; Fr: French; IABP: intra-aortic balloon pump; LV: left ventricular; VA-ECMO: venoarterial extracorporeal membrane oxygenation.

## Data Availability

Data were extracted from corresponding publications of individual trials.
